# Study on isothermal crystallization kinetics of Zr_55.7_Cu_22.4_Ni_7.2_Al_14.7_ bulk amorphous alloy

**DOI:** 10.1038/s41598-022-08848-z

**Published:** 2022-03-24

**Authors:** Weijian Zhang, Pingjun Tao, Yugan Chen, Junfeng Si, Zhenghua Huang, Kunsen Zhu, Yuanzheng Yang

**Affiliations:** 1 School of Materials and Energy, Guangdong University of Technology, Guangzhou, 510006 People’s Republic of China; 2 Guangdong Kiatest Technology Co., LTD, Guangzhou, 510700 People’s Republic of China; 3 Guangdong - Hong Kong Joint Research and Development Center On Advanced Manufacturing Technology for Light Alloys, Institute of New Materials, Guangdong Academy of Sciences, Guangzhou, 510650 People’s Republic of China

**Keywords:** Materials science, Materials for energy and catalysis

## Abstract

Zr_55.7_Cu_22.4_Ni_7.2_Al_14.7_ alloy bars were prepared by copper mold suction casting, and the structure and thermodynamic properties of the prepared specimens were characterized by X-ray diffractometer and differential scanning calorimeter. Furthermore, isothermal crystallization mechanism and crystallization activation energy were investigated by Johnson–Mehl–Avrami model and Arrhenius equation. The results show that the structure of the specimen is amorphous. The glass transition temperature (*T*_*g*_), onset crystallization temperature (*T*_*x*_) and crystallization peak temperature (*T*_*p*_) are 713 K, 779 K, and 781 K, respectively, and supercooled liquid region ($\Delta T\, = \,T_{x} - T_{g}$) is 66 K. The crystallization incubation times are shortened and the crystallization transformation rates increase with temperature rising. As the crystallization volume fraction increases, the crystallization nucleation rate shows a trend of first increasing and subsequently decreasing, while the activation energy decreases monotonously, in the same time, the crystallization behavior transforms from interface-controlled to diffusion-controlled growth. The thermal stability of amorphous alloy is also discussed.

## Introduction

Amorphous alloys are new amorphous metallic glass materials with excellent mechanical, physical and chemical properties of both general metals and glass, made by modern rapid solidification metallurgical techniques. Amorphous alloys, also known as metallic glasses or liquid metals, are composed of a short-range ordered, long-range disordered glassy structure with a more uniform structure and composition than crystalline alloys^1–3^. However, the structure of amorphous alloys, which is in a sub-stable state in thermodynamics, will transform into crystalline structure at a certain temperature or long enough service time, i.e. crystallization will occur^4,5^. This structural transformation will affect various properties of amorphous alloys, such as mechanical and electrochemical properties, thereby affecting their practical applications^6–8^. On the one hand, the appearance of excessive crystalline phases in amorphous alloys will reduce their mechanical properties^9,10^, on the other hand, the performances of amorphous alloys materials will be improved when there are nanocrystals with appropriate size in amorphous alloys^11^.

In recent years, Zr-based bulk amorphous alloys have received extensive attention due to their excellent amorphous forming ability and excellent thermoplastic forming ability^12–15^. The improvement of thermoplastic processing performances by inhibiting the crack expansion and avoiding crystallization during thermoplastic processing of Zr-based bulk amorphous alloy^16,17^, and sought to enhance the thermal stability of amorphous alloys by adding a small amount of rare earth elements to improve the product properties after thermoplastic processing^18,19^. The thermal stability, crystallization kinetics of amorphous alloys are related to thermoplastic forming ability and hot workability of bulk amorphous alloys^20–23^. Although there has been a lot of research work in this area, there are still many issues that are still unclear. In this paper, Zr_55.7_Cu_22.4_Ni_7.2_Al_14.7_ (at.%) alloy specimens were prepared by copper mold suction casting method. The isothermal crystallization kinetics was studied by X-ray diffraction (XRD) and differential scanning calorimetry (DSC), in order to further understand the crystallization mechanism, local Avrami exponent and crystallization activation energy of amorphous alloy. It will lay a theoretical foundation for thermoplastic processing and engineering application of bulk amorphous alloys.

## Experimental

The ingots of Zr_55.7_Cu_22.4_Ni_7.2_Al_14.7_ (at.%) alloy were prepared under a Ti-gettered purified argon atmosphere in a WK-II non-consumable vacuum arc melting furnace using a mixture of high purity metals consisting of Zr (99.96 wt.%), Cu (99.98 wt.%), Ni (99.9 wt.%) and Al (99.97 wt.%). Each alloy ingot was re-melted at least four times for compositional homogeneity. Alloy ingots were re-melted and suction cast into water-cooled copper mold to obtain alloy robs with a diameter of 3 mm. The prepared specimens were characterized by XRD (D/MAX-Ultima IV, Cu-K_α_ radiation) with a wavelength (*λ*) of 1.54056 Å, and DSC (204 F1) in a high purity argon atmosphere. The specimen was heated from room temperature to 873 K at a heating rate of 20 K/min to determine its thermodynamic parameters. The isothermal crystallization kinetic parameters were obtained by heating to a series of temperature points (723 K, 733 K, 743 K, 748 K, 753 K, and 758 K) during supercooled liquid region (SLR) under a heating rate of 120 K/min and then held for an hour using DSC Q2000. The isothermal crystallization behavior and crystallization activation energy of the alloy specimens were analyzed using the Johnson–Mehl–Avrami model and the Arrhenius equation to obtain an evaluation of the thermal stability of amorphous alloy.

The functional relationship between the crystallization volume fraction *x*(*t*) and the time *t* in isothermal crystallization process can be expressed by the Johnson–Mehl–Avrami (JMA)^24^ equation: $x\left( t \right)\, = \,1 - exp\{ - [k(t - \tau )]^{n} \}$. Where $k\, = \,k_{0} exp\left( { - E/RT} \right)$ is the reaction rate constant, *E* is the activation energy, *k*_*0*_ is the constant, *R* is the molar gas constant, *τ* is the incubation time, and *n* is the Avrami exponent (which can reflect the crystallization mechanism of nucleation and nucleus growth during the crystallization process). The logarithm of both sides of the JMA equation can be obtained as follows^12^: $ln\left\{ { - ln\left[ {1 - x\left( t \right)} \right]} \right\}\, = \,n \, lnk\, + \,n \, ln(t - \tau )$.

## Results and discussion

### Identification of alloy specimen structure

**Figure 1:**
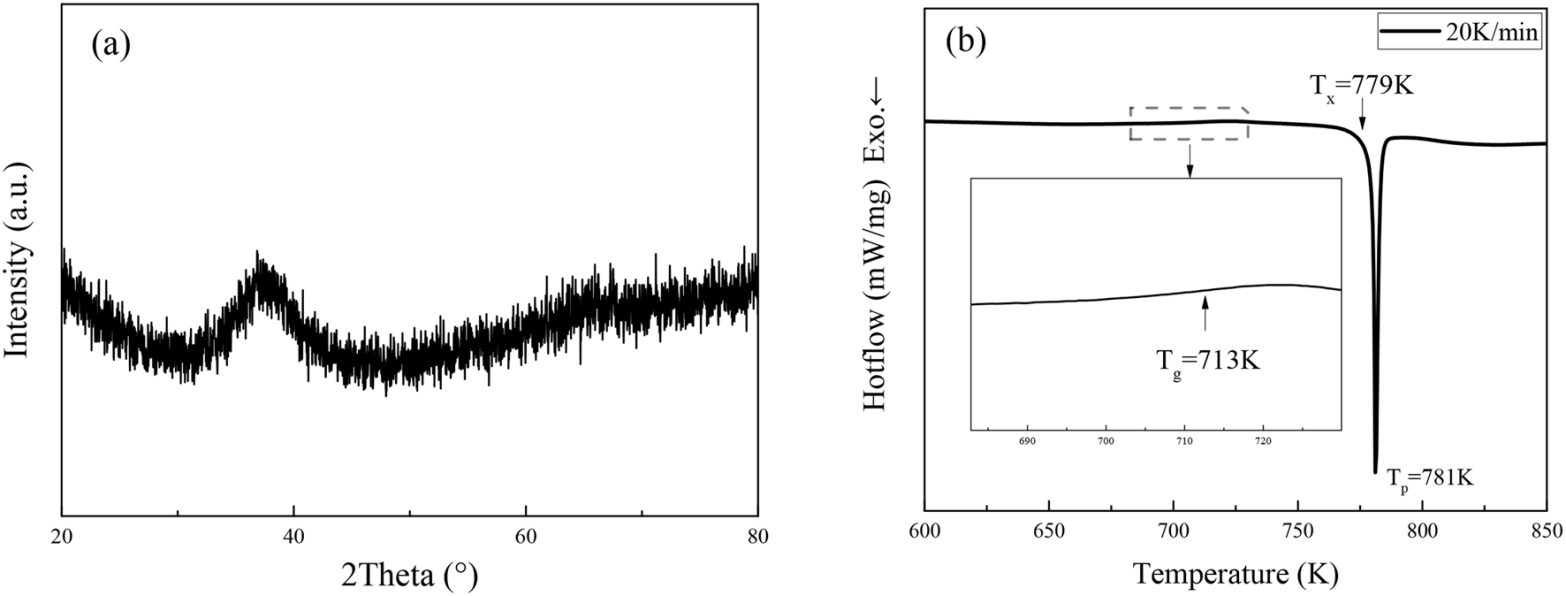
(**a**) XRD pattern and (**b**) DSC scan of Zr_55.7_Cu_22.4_Ni_7.2_Al_14.7_ alloy specimen.

Figure 1a shows XRD pattern of the prepared Zr_55.7_Cu_22.4_Ni_7.2_Al_14.7_ alloy specimen. It can be seen that the diffraction angle (2 theta) is between 32° and 42°, and there is a diffuse scattering peak without sharp crystal diffraction peak, which indicates that the structure of the alloy specimen is amorphous.

### Characteristic temperature of Zr_55.7_Cu_22.4_Ni_7.2_Al_14.7_ bulk amorphous alloy

The DSC scan curve of Zr_55.7_Cu_22.4_Ni_7.2_Al_14.7_ bulk amorphous alloy specimen at a heating rate of 20 K/min is shown in Fig. 1b. The curve shows a single exothermic peak, which is the exothermic reaction of the specimen after the supercooled liquid region (SLR). The glass transition temperature (*T*_*g*_) of this amorphous alloy is 713 K, the onset of crystallization temperature (*T*_*x*_) is 779 K, the crystallization peak temperature (*T*_*p*_) is 781 K, and the SLR is $\Delta T\, = \,T_{x} - T_{g} \, = \,{66}\; {\text{K}}.$ It indicates that the bulk amorphous alloy has a wide SLR, which is extremely beneficial to thermoplastic processing^8^.

### Kinetic analysis of isothermal crystallization

**Figure 2:**
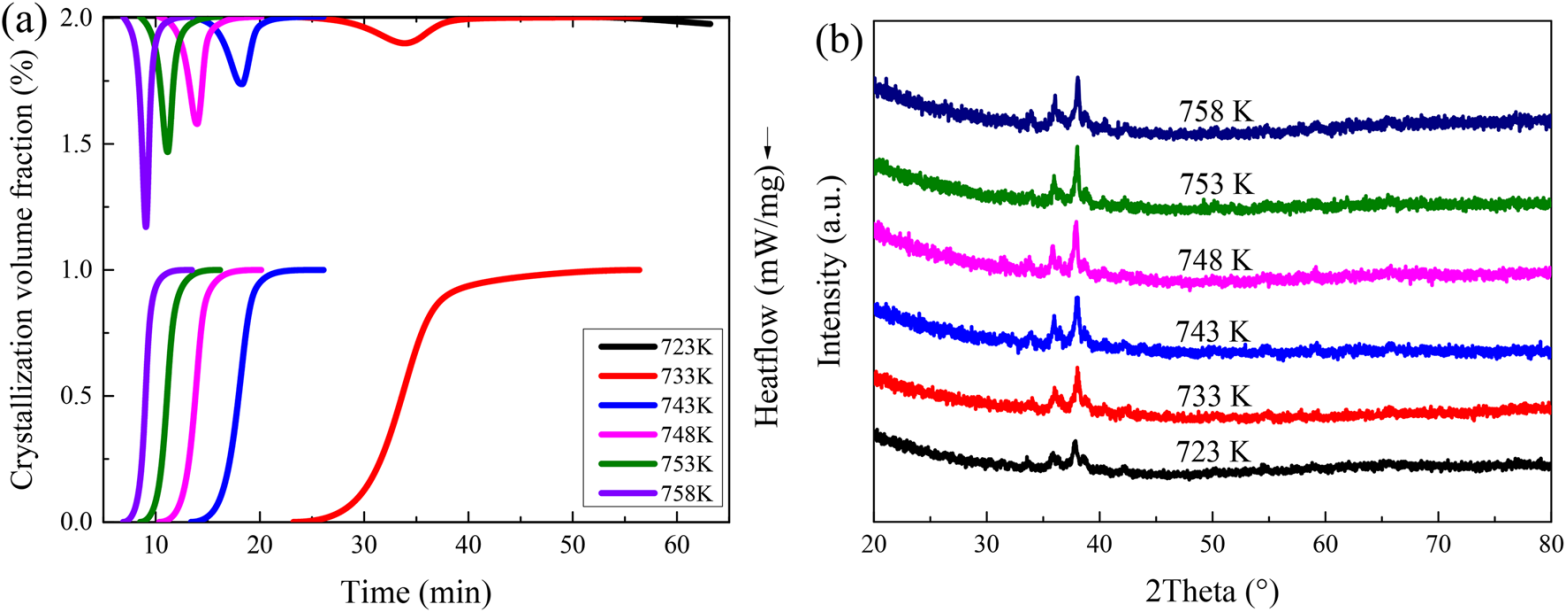
(**a**) Isothermal DSC scans at the top and the crystallization volume fraction at the bottom, (**b**) XRD patterns of the specimens after annealing at the given temperatures.

The DSC curves of isothermal crystallization measurements of alloy specimens at 723 K, 733 K, 743 K, 748 K, 753 K, and 758 K, respectively, are shown in Fig. 2a at the top. Except for the test curve at 723 K, each curve shows a complete exothermic peak, and the appearance time of the crystallization peak decreases with increasing temperature, and the width of the crystallization peak becomes narrower and gradually sharp. The result expresses that heating at a constant temperature above 723 K for an hour, the time required for the alloy specimen to complete the crystalline transformation shortens as the holding temperature rises. XRD patterns of the specimens after annealing at the given temperatures are shown in Fig. 2b. All specimens exhibit obvious crystal diffraction peaks of crystallized phase after annealing at the given temperatures, but the intensity of diffraction peaks is lower for the specimen annealed at 723 K. It indicates that all the alloy specimens have been fully crystallized within the annealing time, except for the specimen annealed at 723 K where partial crystallization occurred. It is consistent with the analytical results of isothermal crystallization DSC.

The heating time *t* and isothermal crystallization volume fraction *x*(*t*) can be calculated by $x\left(t\right)=[{\int }_{{t}_{0}}^{t}(d{H}_{c}/dt)dt]/[{\int }_{{t}_{0}}^{{t}_{\infty }}(d{H}_{c}/dt)dt]={A}_{t}/{A}_{\infty }$^12,18,25^. Where *A*_*t*_ denotes the area of the exothermic peak between the onset of crystallization time *t*_*0*_ and time *t*, *H*_*c*_ is the value of time *t* corresponding to the heat flow, and *A*_*∞*_ denotes the total area of the crystallization exothermic peak.

The relationship between the crystallization volume fraction *x* and time *t* for Zr_55.7_Cu_22.4_Ni_7.2_Al_14.7_ bulk amorphous alloy at different temperatures is presented in Fig. 2a at the bottom. All the curves show S-shape (sigmoidal) with the increasing annealing time, and the S-shape becomes steeper and steeper as the temperature increases, implying that the crystallization rates are increased sharply with the increase of annealing temperature. In the initial stage of crystallization, the crystallization rate is slow, due to crystal nucleation takes a long time to break through the energy barrier. Subsequently, the crystallization rate is extremely fast, because a large number of nuclei are accumulated inside the specimen in the initial stage of crystallization and the nuclei grow rapidly by subsequently, and the volume content of crystals inside the specimen rises suddenly. At the end of crystallization, the nucleation rate drops quickly with the annealing time increases. The amorphous sample structure is almost transformed into a crystalline structure^20,26,27^.

**Figure 3:**
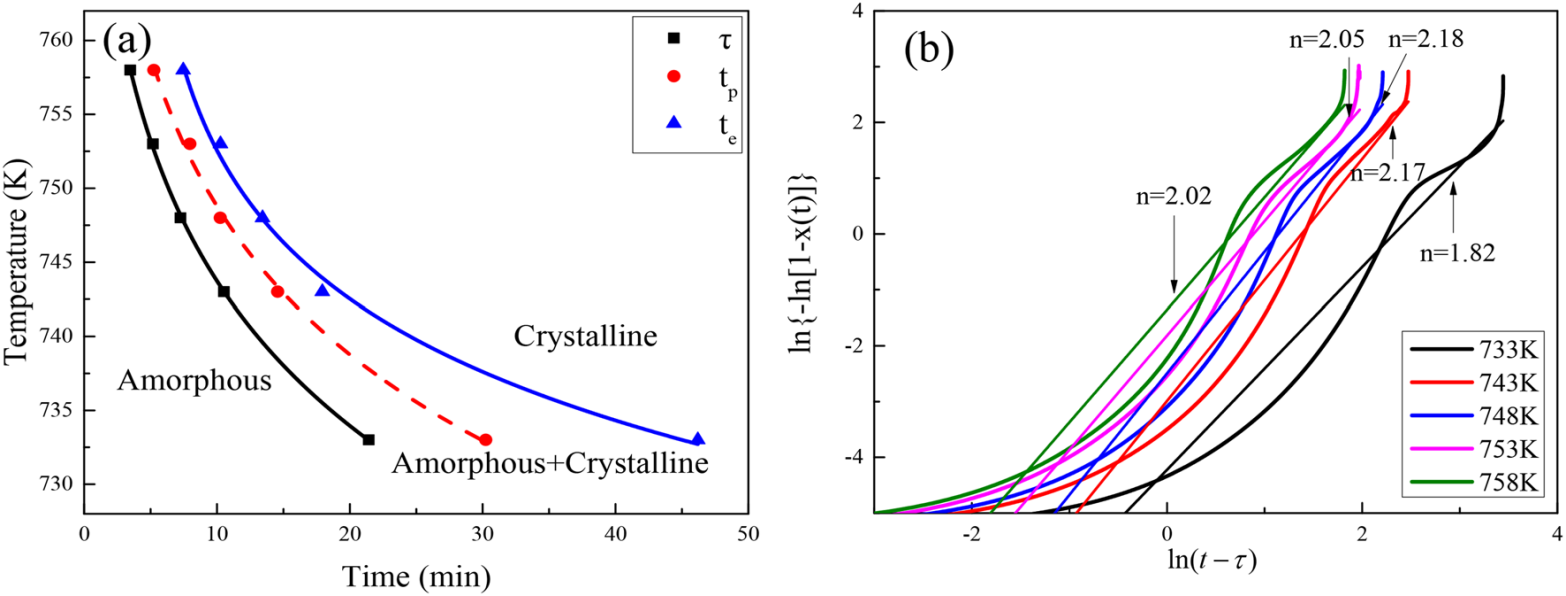
(**a**) Isothermal TTT curves and (**b**) JMA curves at different temperatures.

Figure 3a shows the Time–Temperature-Transformation (TTT) fitting curves^19,28^ of the alloy specimens. Start, 50% fraction and end of crystallization phase transformations correspond to the crystallization incubation time *τ*, the peak crystallization time *t*_*p*_, and the end of crystallization time *t*_*e*_, respectively. At the bottom left of the curve is the amorphous region, where crystallization of amorphous alloy begins after crossing the onset of crystallization transition curve, and at the top right is the crystalline region where crystallization has been completed. As the annealing temperature increases, the crystallization incubation time *τ* is gradually reduced, and the time required for the specimen to complete crystallization is also significantly shortened. This is because atoms can get more energy to accelerate the fluctuation of internal atomic concentration at higher annealing temperatures, making the atomic motion more active and increasing the atomic mobility, which leads to rapid crystallization of the internal structure of bulk amorphous alloy^19^.

As shown in Fig. 3b, the JMA curves of the specimens at different temperatures are obtained by plotting $ln\left\{ { - ln\left[ {1 - x\left( t \right)} \right]} \right\}$ versus $ln\left( {t - \tau } \right)$. A linear fit to the JMA curve yields the slope and intercept of the line, with the slope corresponding to the Avrami exponent *n* and the intercept to the reaction rate constant *k*. The Avrami exponent values are distributed between 1.8 and 2.2, indicating that the crystallization mechanism is similar at different temperatures^29^. The reaction rate constant *k* increases with rising temperature, revealing that the alloy is easier to crystallize at higher temperatures, due to the fact that the diffusion ability of internal atoms increases with rising temperature, and the diffusion of atoms facilitates structural ordering, so the crystallization reaction rate of bulk amorphous alloy increases with rising temperature^20^.

The crystallization behavior of amorphous alloys will show a certain variation pattern with the change of crystallization volume fraction *x*. During the crystallization of amorphous alloys, metastable phases may appear, and will transform into stable phases or transform into other sub-stable phases first and subsequently transform into stable phases^23,27^. Hence, Calka^30^ proposed that the mechanism of nucleation and growth behavior can be characterized in more detail by analyzing the variation pattern of the local Avrami exponent. The local Avrami exponent *n*(*x*) can be expressed by the expression: *n*(*x*) = {*əln*[−*ln*(*1−x*)]*/əln*(*t−τ*)}. Ranganthan proposed that the local Avrami exponent *n* can be expressed by the formula^31,32^: *n* = *a* + *bp.* Where *a* is the nucleation mechanism parameter; *b* is the dimension of crystal nucleus growth; *p* is the growth mechanism parameter. And *a*, *b* and *p* content the following definitions: *a* = 0 indicates that the nucleation rate is zero; *a* = 1 indicates that the nucleation rate is constant; *a* > 1 indicates that the nucleation rate increases with time; *a* < 1 indicates that the nucleation rate decreases with time. *b* = 1, 2, 3 respectively represent one-dimensional, two-dimensional, and three-dimensional growth. *p* = 0.5 indicates the growth of diffusion-controlled; *p* = 1.0 indicates the growth of interface-controlled^20^.

**Figure 4:**
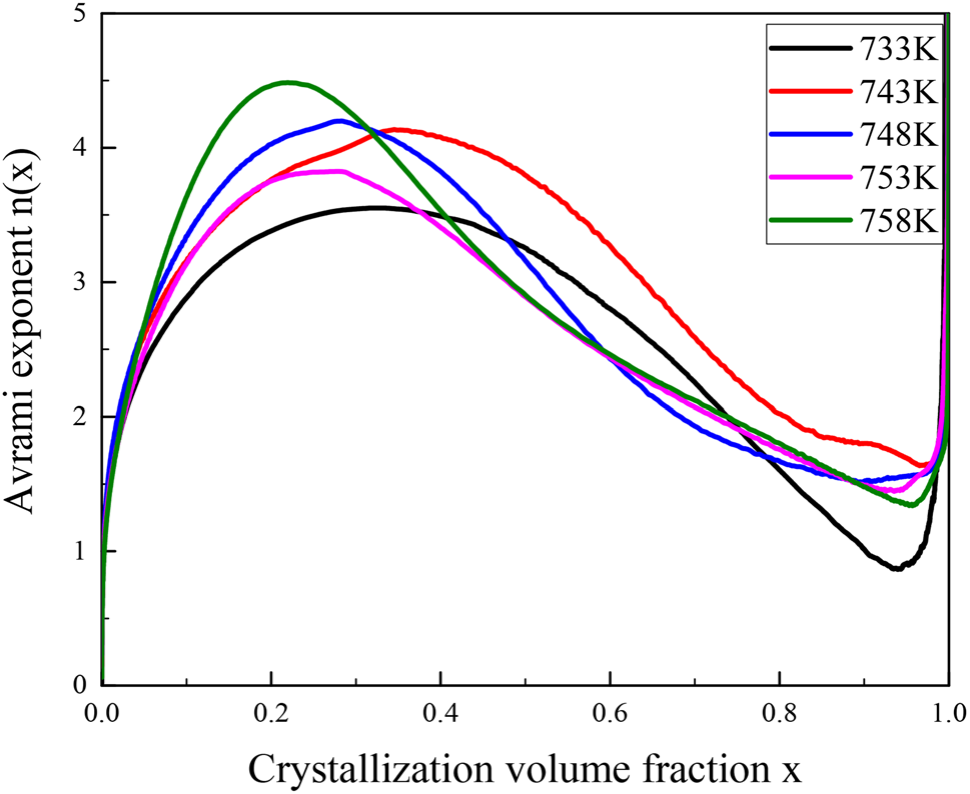
Curves between local Avrami exponent and crystallization volume fraction.

Figure 4 shows the relationship curves between the local Avrami exponent *n*(*x*) and the crystallization volume fraction *x* of the alloy. Each curve has a similar trend with respect to the crystallization volume fraction *x*, indicating that the *n*(*x*) does not vary much under the influence of different temperatures. At the same temperature, *n*(*x*) varies significantly with *x* increasing, which exposes that the crystallization mechanism of bulk amorphous alloy is different at different crystallization stages^20^.

The crystallization behavior of Zr_55.7_Cu_22.4_Ni_7.2_Al_14.7_ alloy specimen at a temperature of 758 K is analyzed as follows: the Avrami exponent *n* increases with the crystallization volume fraction to 4.5 and drops sharply to 1.5 by subsequently, indicating that the nucleation behavior of the crystal phase in this alloy is first interface-controlled and then transform to a diffusion-controlled growth behavior^20,23^. In the initial stage of crystallization, the value of *n* is quite tiny, indicating that nucleation rate is pretty slow, because there are a large number of supercooled liquid without nucleation in the specimen, and there are a large amount of short-range ordered atomic clusters, which assume the role of supercritical nuclei to discourage the formation of new crystal nuclei^28^. When 10% < *x* < 37%, *n* rises to 4.5 and then reduces to 4, indicating that the alloy crystallization nucleation rate is accelerated with the increase of *x*. The crystallization behavior is controlled by the interface-controlled three-dimensional growth mechanism. This is because the previous nucleation growth in amorphous alloy will cause the composition change of the surrounding matrix^12^. Thus, it promotes the formation of new nuclei. When 37% < *x* < 50%, n decreases from 4 to 3, indicating that the crystallization nucleation rate decreases with *x*. Its nucleation growth behavior is still controlled by interface-controlled, and both supercritical nuclei and new nuclei generated demand for undergoing atomic rearrangement, however, the tight and irregular structure of atomic arrangement of multi-component bulk amorphous alloy makes atomic diffusion difficult^28^, which blocks the generation of new crystal nuclei. When 50% < *x* < 95%, *n* reduces to 1.5, indicating that the nucleation growth dominates in this stage and the nucleation growth behavior transforms to diffusion-controlled^20^. That is why the internal crystallization nucleation rate decreases. When *x* > 95%, the data at this stage will not be discussed on account of the large error of *n* value calculated by the JMA model. Therefore, heat treatment or thermal processing should be carried out at a lower temperature with SLR, while the heat treatment time should be reduced accordingly when the thermal processing temperature rises^8^.

In addition to the Avrami exponent evaluation, the crystallization behavior of amorphous alloys can also be characterized by the change of crystallization activation energy. The activation energy of crystallization is calculated by Arrhenius equation^12,33^: *t*(*x*) = *t*_*0*_*exp*(*E*_*a*_*/RT*). Where *t*(*x*) is the time corresponding to crystallization volume fraction *x*, *t*_*0*_ is a constant, *E*_*a*_ is the crystallization activation energy, *R* is the molar gas constant, and *T* is the corresponding isothermal crystallization temperature.

**Figure 5:**
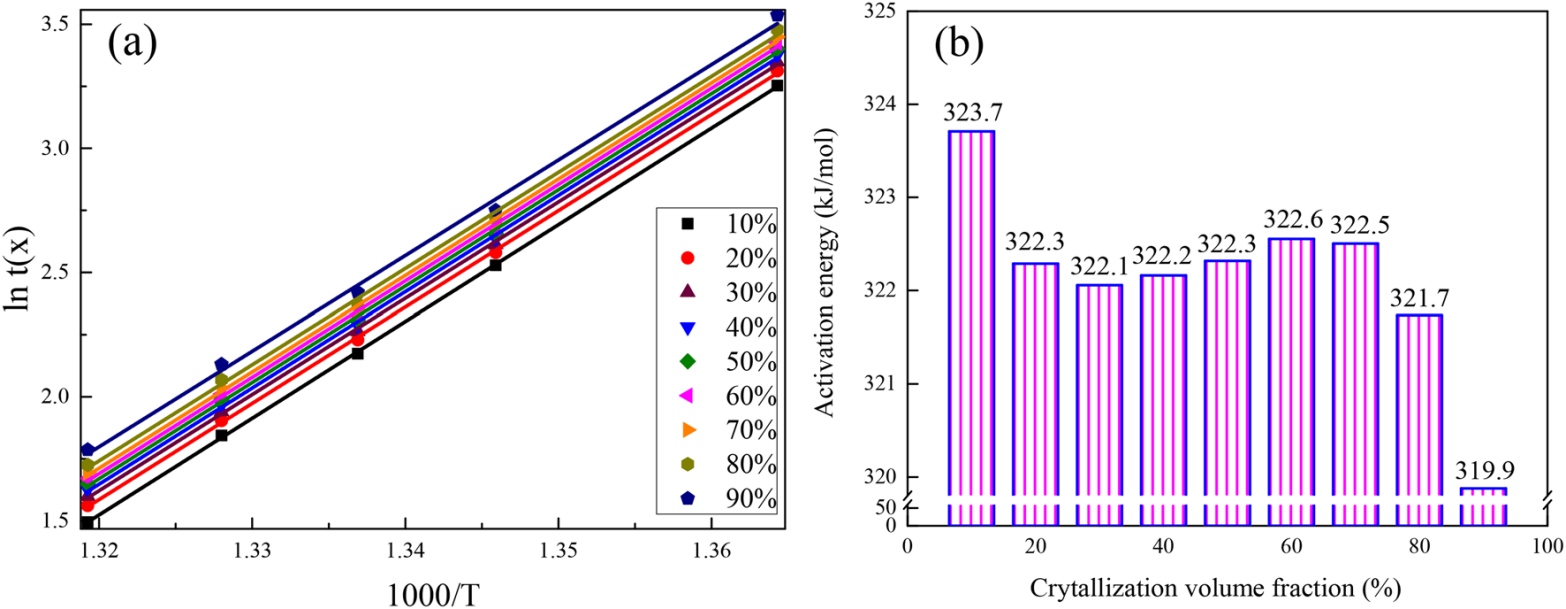
(**a**) Plots of *ln t*(*x*) versus 1000*/T* and (**b**) activation energy *E*_*a*_ versus crystallization volume fractions.

Figure 5a is obtained by plotting *ln t*(*x*) versus *1000/T* and linear fitting the data under each crystallization volume fraction. The slope obtained by linear fitting can be calculated by the Arrhenius equation to acquire the relationship curve between the crystallization activation energy and the crystallization volume fraction *x*^8^, as shown in Fig. 5b. The crystallization activation energy of bulk amorphous alloy shows a decreasing trend as *x* increases. In the initial stage of crystallization, the higher activation energy of crystallization indicates that the alloy needs to break through a higher energy barrier for crystallization^20^. The crystallization activation energy decreases rapidly when 10% < *x* < 20%, implying that the crystallization behavior becomes easier after the initiation of crystallization. When 20% < *x* < 70%, the crystallization activation energy is basically stable at 322.3 kJ/mol, indicating that the crystallization reaction is comparatively stable in this process. When *x* > 70%, the crystallization activation energy reduces rapidly, indicating that the new crystal nucleation rate reduces rapidly in the ultimate stage of crystallization, the crystallization behavior transforms into crystals growth, and the residual amorphous matrix volume content inside alloy gradually decreases^12^. Therefore, it is difficult for atoms in amorphous alloys to advance nucleation, and the structure of amorphous alloy becomes comparatively stable after crystallization behavior^34^. This is basically consistent with the analysis result of Fig. 4. The average crystallization activation energy of this bulk amorphous alloy is 322.1 kJ/mol, which is higher than the other components’ in the same system of Zr–Cu–Ni–Al^12^. Thus, Zr_55.7_Cu_22.4_Ni_7.2_Al_14.7_ bulk amorphous alloy has relatively great thermal stability.

## Conclusions

The structure of the prepared Zr_55.7_Cu_22.4_Ni_7.2_Al_14.7_ alloy specimen is amorphous. The glass transition temperature (*T*_*g*_), the onset of crystallization temperature (*T*_*x*_) and the crystallization peak temperature (*T*_*p*_) are 713 K, 779 K, and 781 K, respectively, and the supercooled liquid region (*△T* = *T*_*x*_*−T*_*g*_) is 66 K. The crystallization incubation times are shortened and the crystallization transformation rates increase with the rise of temperature. The Avrami exponents are situated at 1.8 ~ 2.2. As the crystallization volume fraction (*x*) increases, the crystallization nucleation rate shows a trend of first increasing and decreasing by subsequently, while the crystallization activation energy (*E*_*a*_) decreases monotonously. The average crystallization activation energy is 322.1 kJ/mol, in the same time, the crystallization behavior transforms from interface-controlled to diffusion-controlled growth. The Zr_55.7_Cu_22.4_Ni_7.2_Al_14.7_ amorphous alloy has a great thermal stability.
